# A qualitative study exploring newborn care behaviours after home births in rural Ethiopia: implications for adoption of essential interventions for saving newborn lives

**DOI:** 10.1186/s12884-014-0412-0

**Published:** 2014-12-12

**Authors:** Mihretab Melesse Salasibew, Suzanne Filteau, Tanya Marchant

**Affiliations:** London School of Hygiene and Tropical Medicine, Keppel Street, London, WC1E 7HT UK

**Keywords:** Newborn care, Newborn behaviours, Newborn practices, Essential interventions, Delayed bathing, Cord tying, Thermal care, Breastfeeding

## Abstract

**Background:**

Ethiopia is among seven high-mortality countries which have achieved the fourth millennium development goal with over two-thirds reduction in under-five mortality rate. However, the proportion of neonatal deaths continues to rise and recent studies reported low coverage of the essential interventions saving newborn lives. In the context of low uptake of health facility delivery, it is relevant to explore routine practices during home deliveries and, in this study, we explored the sequence of immediate newborn care practices and associated beliefs following home deliveries in rural communities in Ethiopia.

**Methods:**

Between April-May 2013, we conducted 26 semi-structured interviews and 2 focus group discussions with eligible mothers, as well as a key informant interview with a local expert in traditional newborn care practices in rural Basona woreda (district) near the urban town of Debrebirhan, 120 km from Addis Ababa, Ethiopia.

**Results:**

The most frequently cited sequence of newborn care practices reported by mothers with home deliveries in the rural Basona woreda was to tie the cord, immediately bath then dry the newborn, practice ‘Lanka mansat’ (local traditional practice on newborns), give pre-lacteal feeding and then initiate breastfeeding. For ‘Lanka mansat’, the traditional birth attendant applies mild pressure inside the baby’s mouth on the soft palate using her index finger. This is performed believing that the baby will have ‘better voice’ and ‘speak clearly’ later in life.

**Conclusion:**

Coverage figures fail to tell the whole story as to why some essential interventions are not practiced and, in this study, we identified established norms or routines within the rural communities that determine the sequence of newborn care practices following home births. This might explain why some mothers delay initiation of breastfeeding and implementation of other recommended essential interventions saving newborn lives. An in-depth understanding of established routines is necessary, and community health extension workers require further training and negotiation skills in order to change the behaviour of mothers in practicing essential interventions while respecting local values and norms within the communities.

## Background

Globally, a marked 41% reduction has been recorded in under-five mortality: from 87 deaths per 1,000 live births in 1990 to 51 per 1,000 in 2011. Although sub-Saharan Africa still has the highest mortality rates in the world, the region doubled its annual rate of mortality reduction from 1.5 per cent a year between 1990 to 2000 to 3.1 per cent a year between 2000 to 2011. However, similar improvements in newborn survival (first 28 days of life) have not been observed, and therefore the percent of all under-five deaths that occur in the newborn period increased from 36 percent in 1990 to 43 percent in 2011 [1]. Essential interventions immediately after birth such as thermal care (immediate drying and delayed bathing), early initiation of breastfeeding (within one hour) as well as hygienic cord and skin care are recommended to save newborn lives [2]. It has been estimated that if coverage was universal, exclusive breastfeeding, thermal care and cord care could save up to 13%, 2% and 4% of all under-five deaths respectively [3].

Between 1990 and 2010, seven high-mortality countries (Bangladesh, Ethiopia, Liberia, Malawi, Nepal, United Republic of Tanzania and Timor-Leste) had reduced their under-five mortality by two-thirds or more. Ethiopia recoded a 67% reduction in under-five mortality; hence it already achieved the fourth millennium development goal [4]. However, in 2012 a large population-based survey in Ethiopia [5] reported that only 50% of newborns had early initiation of breastfeeding, 41% had clean cord care, 43% immediate drying and 47% delayed bathing. These sub-optimal coverage estimates were consistent with other surveys from Ethiopia [6] and elsewhere in Africa [7-9] and Asia [10-14].

The potential of essential interventions to save newborn Ethiopian lives, therefore, has not yet been realized and there is a need for continued efforts to increase coverage especially in rural areas where access to health services and information about newborn care is limited. Nine of every ten mothers in Ethiopia deliver at home [15] and, in the context of low uptake of health facility delivery, it is relevant to explore routine immediate newborn care practices that follow the birth of a baby at home within rural communities in Ethiopia. Therefore, in this study, we report on the sequence of newborn care practices and associated beliefs following home births in rural Ethiopia.

## Methods

We used qualitative methods to gain an in-depth understanding about newborn care practices and associated beliefs in rural Ethiopia.

### Setting

A breastfeeding measurement study [16] was conducted between April and May 2013 in Basona (rural) and Debrebirhan (urban) woredas (districts) in Ethiopia which are located in Semen Shewa Zone (sub-region) in Amhara regional state and 120 km away from Addis Ababa, the capital city of Ethiopia. These districts were chosen for convenience, being close to Debrebirhan University where a research assistant facilitated the data collection process. Basona woreda has a total population of 120, 930, is predominantly rural and women aged 15–49 years old constitute one fifth of its total population [17]. As part of the breastfeeding measurement study, we explored the sequence of immediate newborn care practices and associated beliefs following home deliveries in the rural Basona woreda.

### Study participants

Mothers aged 15–49 years who had at least one live home birth in the last two years, and who had ever breastfed that child, and who were resident in the rural Basona woreda, were participants of the study. Health extension workers, who keep a list of women aged 15–49 years old in their catchment area, assisted during recruitment by identifying and contacting eligible mothers in their homes. Mothers who agreed to participate in the study were then invited for interview in a venue within their community in a room provided by the local government administrative unit.

### Data collection

Data was collected using 26 semi-structured interviews, 2 focus group discussions (9 participants in each group) and a key informant interview with a local expert on traditional newborn care practices.

During the semi-structured interviews, we asked mothers an open-ended question about the context within which they initiated breastfeeding and all events and newborn care practices they remember taking place during that home delivery. Stories of mothers covered a range of topics including who assisted them, which family member was around, whether they had difficulty with labour, whether the baby was given a bath, dried, breastfed etc. At the end of their story, we probed mothers to establish the sequence of newborn care practices or the order in which those events happened [Table 1: topic guides for semi-structured interviews and focus group discussions].

**Table 1:** **Topic guides for semi-structured interviews and focus group discussions**

**Semi-structured interviews**	**Focus group discussions**
**Question**	**Question**
I try not to interrupt you; so please feel free to tell me everything you remember about this birth and the context when it happened starting from when you went to labour, newborn care your baby received and other events that followed immediately after the birth of [name].	During the interviews, you told me about the context within which you initiated breastfeeding and all events and newborn care practices you remember taking place following the birth [name]. In this focus group discussion, I would like to discuss some of these practices further in order to understand how common these practices are within your community and more details about how each newborn care behavior is practiced and underlying beliefs, if any
**Probes**	**Discussion points**
Who attended the birth?	Cord care
Cord care = what was used to cut and tie the cord? Did you apply anything on it?	Immediate bathing & drying
Thermal care = was the baby given immediate bath and dried?	
Breastfeeding initiation = when did you start breastfeeding your baby, did you give baby anything other than breastmilk soon after birth?	Lanka mansat
Any other newborn care practice you would like to mention please?	Pre-lacteal feeding and early initiation of breastfeeding

In order to gather views of mothers from diverse backgrounds, we categorized eligible mothers based on two key characteristics: level of education (illiterate or ≥ primary level education) and parity (first time mother or with more than one child). We continued to interview mothers in one category until saturation was reached and before we moved on to the second category.

On days when a sufficient number of women attended, mothers interviewed were then invited to stay to attend the focus group discussions. This way, we managed to do 2 focus group discussions on 2 separate days with 18 mothers (9 in each focus group discussion). In these discussions we further explored whether the sequence of newborn care practices reported during semi-structured interviews were regarded as a norm within the community at large.

All interviews and facilitated focus group discussions were conducted in the Amharic language. The number of interviews conducted was determined by reaching the saturation point, i.e. when there were no more new emerging views. Interviews and focus group discussions were conducted with only mothers and the interviewer present and were recorded using a digital-audio recorder.

### Data analysis

All interviews and focus group discussions were transcribed verbatim directly from Amharic audios into English transcripts initially by a lecturer from Debrebirhan University and then by the principal investigator. Data was analysed deductively using the framework analysis approach [18]. Using qualitative data analysis software package NVIVO version 10, each transcript was carefully screened and coded. These codes were in turn grouped into major themes representing reported newborn care practices such as bathing, cord care or breastfeeding initiation. For each mother, identified themes were outlined in a chronological order starting from the first newborn care practice immediately after birth to the time when breastfeeding was initiated. Finally, the most frequently cited sequence of newborn care practices was identified among all mothers interviewed in the study.

### Ethics

The study received ethical approval from research ethics committee at the London School of Hygiene and Tropical Medicine in October 2012 and in Ethiopia from the National Research Ethics Committee under the Ministry of Science and Technology in April 2013. Further support letters were also obtained from Ethiopian Ministry of Health, Debrebirhan University and Basona woreda health bureau. All eligible mothers gave written informed consent or thumb print prior to interviews.

## Results

**Figure 1:**
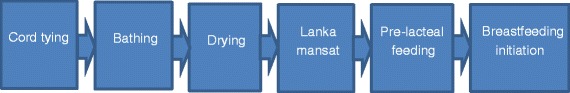
**Sequence of newborn care practices for home deliveries in rural Basona woreda.**

Although there were some differences between maternal reports about which newborn care behaviours they practiced, if at all, the most frequently cited sequence of newborn care practices among mothers with home deliveries in the rural Basona woreda was to tie the cord, immediately bath then dry the newborn, practise ‘Lanka mansat’ (local traditional practice on newborns and described below), give pre-lacteal feeding and then initiate breastfeeding (Figure 1: Sequence of newborn care practices for home deliveries in rural Basona woreda).

A. Cord care

Almost all mothers interviewed said they used clean blades for cutting the cord, tied the cord using a string or thread and did not apply anything on the cord during the process of drying.

*“They [Traditional birth attendants (TBAs)] cut the placenta using clean blades and tied the cord there and then with a thread …we don’t apply anything on the cord”* [First time mother, literate]

B. Bathing and drying

Immediate bathing was a common practice and mothers reported that either the TBA or families who attended the birth give the baby a bath immediately after birth, dried and wrapped the baby in a clean towel before passing it to another family member or a friend to hug the baby for a while.

*“Soon after birth, she gave the baby a bath, wrapped her with a towel; she hugged her a bit and then gave me back to breastfeed”* [Mother of two, literate]

*“…I gave birth on the street on my way to hospital, we returned back home and my family gave a bath to the baby while I sat down waiting for the placenta to come…”* [First time mother, literate]

However, some mothers claimed that they had had health education from health professionals and community health extension workers about delaying bath. As a result, they no longer bathed newborns immediately after birth.

*“She only dried and wrapped the baby in a clean towel soon after birth, that’s the first thing she did. She said we don’t bath babies any more. I also heard this in trainings from the health professionals before”* [Mother of 5, illiterate]

In the focus group discussions, all mothers agreed that immediate bathing of newborn babies was the norm when a mother gives birth at home. A few mothers, who argued for changing this behaviour, made reference to the information they heard from community health extension workers or recent health education sessions they attended about delaying bathing. However, there were discrepancies as to how long these mothers thought bathing should be delayed for, with responses ranging from 2 hours to 24 hours after birth. Therefore, despite their awareness about delaying bathing, mothers were not clear about for how long bathing babies should be delayed.

C. Lanka Mansat

This is not part of the essential interventions [2] and is not a recommended practice to save newborn lives. However, it was reported that while the mother waits for the placenta to be delivered, the TBAs or family members who attended the birth perform a traditional practice on the newborn baby which they refer to as ‘Lanka Mansat’. ‘Lanka mansat’ is a local traditional practice whereby the TBA applies mild pressure inside the baby’s mouth on the soft palate using her index finger which is made wet using a mixture of warm water and a local herb called ‘ersho’ or honey. Some mothers preferred to do ‘Lanka mansat’ just after tying the cord and before immediate bathing.

*“After birth, she picked the baby up and applied Ersho [local herb] to his Lanka [soft palate]. She then gave the baby a mixture of water and sugar using a spoon [pre-lacteal feeding] before letting him sleep a bit. The placenta came out and after they gave me some food to eat, I got my baby to breastfeed”* [Mother of 2, literate]

*“…no one eats meal in the house until ‘Lanka Mansat’ is done and the baby is given bath, dried and wrapped up in a towel”* [Mother of 2, illiterate]

*“…the way they [TBAs] do it is, first they put some honey in to a small cup and then immerse their index finger in to the cup and apply mild pressure in the baby’s mouth [soft palate] using their index finger lifting it [soft palate] upwards …”* [Mother of 4, illiterate]

Mothers believed this helps the baby to have a ‘better voice’ and ‘be able to speak clearly’ when he or she gets older.

*“I don’t know how it works but in our tradition people believe children will have better voice when they grow up and can be listened to from distance if they had ‘Lanka mansat’ done”* [Mother of 2, literate]

*“…because, if ‘Lanka mansat’ is not done, babies cannot talk properly when they grow up”* [Mother of 3, literate]

A total of 16 out of 26 mothers confirmed that their last child had Lanka mansat performed, but all multiparous mothers said this was a practice performed with their older children. In the focus group discussions, there was a broad consensus among mothers in practising ‘Lanka mansat’ as part of their culture or tradition in their community. A few mothers argued they no longer practise it because of advice from health professionals and community health extension workers about not giving anything to the newborn baby other than breastmilk. However, there was no mention of health workers advising them against practising ‘Lanka Mansat’ specifically. We also noted during these discussions that some mothers strongly believed in the tradition and, despite hearing the advice from the professionals, they were keen to continue practising it.

Findings from the key informant interview with a local expert on traditional newborn care practices revealed that ‘Lanka mansat’ was not listed as one of the traditional practices they identified in the rural Basona woreda.

*“…I am not aware of this; we run a number of projects to raise awareness in harmful traditional newborn care practices common in this area which are female genital mutilation, uvula cutting, milk teeth extraction, marriage before 15 years and marriage by abduction ….we also try to enforce the law on some individuals as some of the harmful traditional practices I mentioned are now illegal…”*

D. Initiation of breastfeeding

Pre-lacteal feeding i.e. feeding babies anything other than breastmilk before the initiation of breastfeeding [19] is common among most mothers who gave birth at home in the rural woreda Basona. Immediately after birth, newborn babies were usually given a mixture of warm water and sugar until the mother was ready to breastfeed regardless of whether that baby had Lanka Mansat done or not.

*“My mother gave the baby warm water with sugar using a spoon [pre-lacteal feeding]. She then gave me the baby after I become stronger and ready to breastfeed”* [Mother of 2, literate]

Timing for initiation of breastfeeding varied from 30 minutes to 7 or 8 hours after birth. However, accuracy in all reports about timing for breastfeeding initiation needs to be interpreted with caution as most mothers, especially those in rural settings, found it difficult to describe time in minutes or hours.

*“I don’t know the time…? It is difficult to say…”* [Mother of 2, illiterate]

*“I gave birth in the morning and breastfed him in the afternoon…”* [Mother of 2, illiterate,]

## Discussion

Beyond reported coverage figures about interventions saving newborn lives, in this study, we provided an in-depth insight into reported behaviours and sequences of immediate newborn care practices during home deliveries in rural Ethiopia. Reported practices in clean cord care appeared to be in line with those recommended as life-saving interventions. However, maternal reports about thermal care and immediate breastfeeding do not currently align with recommended practices, although there was some evidence that health education messages were beginning to change maternal practices.

There are established norms or routines within communities [20-23] and, in this study, we identified the most commonly cited sequence of newborn care practices following home births in the rural Barona woreda in Ethiopia. Almost all mothers in our study reported that they did not apply anything on the cord in the drying process. However, we found that immediate bathing followed by drying of the newborn was the norm, similar to findings in other studies in Ethiopia [6]. Pre-lacteal feeding was reported by almost all mothers we interviewed. All respondents reported breastfeeding their newborn, consistent with evidence that breastfeeding is almost universal in Ethiopia with over 98% of children ever breastfed at some point [15], but we found that initiation of breastfeeding within an hour after birth was problematic for some women. This may be because of challenges when reporting time for breastfeeding initiation [16] but can also be partly explained by competing newborn care practices that families prioritise over immediate breastfeeding.

We also identified a local traditional newborn care practice referred to as ‘Lanka mansat’ which was performed by TBAs or family members during home births. A study in Ethiopia [24] did not list ‘Lanka mansat’ as one of the traditional practices in the area and this was confirmed in the interview with a local expert. To our knowledge, no studies have been conducted to explore and understand more about such traditional newborn care practices. The soft palate is anatomically located at the back of the roof of the mouth and consists of mainly muscle tissues with no bone structure. One of its functions is to assist in making speech sounds and any abnormalities in the soft palate can cause inability to articulate certain sounds [25]. It is this structure on which the TBAs apply mild pressure while performing ‘Lanka mansat’.

In generating this evidence we experienced three important limitations. First, the findings were limited to home births and don’t apply to mothers who gave birth in health facilities. Second, the results represent one rural community in Ethiopia and may not be indicative of practices beyond this study area. Finally, participants of the focus group discussions were the same mothers who attended individual semi-structured interviews and this meant a more limited number of perspectives were included in the study. However, we believe inviting the same mothers who attended individual interviews to also participate in focus group discussions was useful in order to establish (1) whether practices they described individually were also accepted as a norm or a culture within the community at large and (2) how strongly these mothers believe and argue about newborn care practices they mentioned during one-to-one interviews.

## Conclusion

Coverage figures fail to tell the whole story as to why some essential interventions are not practiced – or have low coverage - and, in this study we identified established norms or routines within the rural communities that determine the sequence of newborn care practices following home births. These findings help to explain why some mothers delay initiation of breastfeeding or choose to bath their baby immediately after birth, for example.

The findings have implications to the adoption and implementation of the essential interventions saving newborn lives such as thermal care (immediate drying and delayed bathing), early initiation of breastfeeding (within one hour) as well as hygienic cord and skin care. Therefore, we recommend an in-depth understanding about established routines in immediate newborn care practices within communities prior to designing programs and implementation strategies which aim to promote and increase coverage in the essential interventions. Community health extension workers also require further training and negotiation skills in order to attempt to change the behaviour of mothers, while respecting local values and norms within the communities.
